# Oxidative stress and its biomarkers in systemic lupus erythematosus

**DOI:** 10.1186/1423-0127-21-23

**Published:** 2014-03-17

**Authors:** Dilip Shah, Nidhi Mahajan, Sangita Sah, Swapan K Nath, Bishnuhari Paudyal

**Affiliations:** 1 Center for Translational Medicine, Thomas Jefferson University, Philadelphia, PA 19107, USA; 2 Department of Biochemistry, Panjab University, Chandigarh 160014, India; 3 Department of Biochemistry, JNMC, Belgaum, Karnataka, India; 4 Arthritis and Clinical Immunology Research Program, Oklahoma Medical Research Foundation, Oklahoma City, OK 73104, USA; 5 Department of Radiology, Thomas Jefferson University, Philadelphia, PA 19107, USA

**Keywords:** Oxidative stress, Biomarkers, Antioxidant therapy, Systemic lupus erythematosus

## Abstract

Systemic lupus erythematosus (SLE) is an autoimmune inflammatory disease whose etiology remains largely unknown. The uncontrolled oxidative stress in SLE contributes to functional oxidative modifications of cellular protein, lipid and DNA and consequences of oxidative modification play a crucial role in immunomodulation and trigger autoimmunity. Measurements of oxidative modified protein, lipid and DNA in biological samples from SLE patients may assist in the elucidation of the pathophysiological mechanisms of the oxidative stress-related damage, the prediction of disease prognosis and the selection of adequate treatment in the early stage of disease. Application of these biomarkers in disease may indicate the early effectiveness of the therapy. This review is intended to provide an overview of various reactive oxygen species (ROS) formed during the state of disease and their biomarkers linking with disease. The first part of the review presents biochemistry and pathophysiology of ROS and antioxidant system in disease. The second part of the review discusses the recent development of oxidative stress biomarkers that relates pathogenesis in SLE patients and animal model. Finally, this review also describes the reported clinical trials of antioxidant in the disease that have evaluated the efficacy of antioxidant in the management of disease with ongoing conventional therapy.

## Introduction

Systemic lupus erythematosus is an autoimmune inflammatory disease characterized by the presence of flare of autoantibodies, especially against nuclear components. Although it is believed that the etiology of SLE is multifactorial, including immune dysfunction, genetic, hormonal and environmental, the molecular mechanisms underlying this systemic autoimmune response remain largely unknown. A key issue in the pathogenesis of lupus is how intracellular antigens become exposed and targeted by the immune system [1,2]. In this regard, excessive production of ROS and altered redox state which may cause abnormal activation of apoptosis [3,4], are considered as imperative factors involved in production, expansion of antibody flares and various clinical features in SLE [2]. Several studies have shown the role of ROS in the deregulation of apoptosis and cause increased apoptosis and delayed in clearance of apoptotic bodies. The delay in clearance of apoptotic cells may prolong interaction between ROS and nuclear debris and generate neo-epitopes that subsequently stimulate broad spectrum of autoantibody formation leading to inflammation and organ damage in SLE [5]. The noxious effects of ROS may cause the following consequence: i) shift of intracellular redox (i.e. decrease GSH/GSSH ratio) condition, ii) oxidative modification of lipid, protein and DNA, iii) gene activation of oxidative stress and gene mutation related to antioxidant enzymes.

Considerable genetic studies in SLE patients and animal models implicate the role of oxidative stress in the pathogenesis of SLE. Numerous gene polymorphisms encoding superoxide dismutase (SOD), catalase (CAT), glutathione peroxidase (GPx) and NADPH oxidase encoding gene NCF2 have been identified in association with SLE risk, however some of these associations appear to be ethicity dependent [6]. The Nrf2-Keap1 pathway is important in protection against oxidative stress and inflammation. A strong association between Nrf2-653 G/A polymorphism and the development of nephritis has been reported in Mexican SLE patients [7]. CAT polymorphism (−330CC genotype) is significantly associated with thrombocytopenia, renal manifestations, as well as production of anti-snRNP and anti-Scl-70 antibodies in SLE patients [8]. A member of S-transferase superfamily, GSTM1 may be associated with the production of anti-RO antibodies, suggesting that dysfunction of this gene may be related to the anti-RO autoantibody response or to photosensitivity [9,10].

All biomolecules (lipid, protein and DNA) can be damaged by excessive production of ROS (including ONOO^−^) and may be deleterious and concomitant. Product of these cascades of oxidative modification can be detected in biological fluid and their abundance correlates with disease activity and organ damage in SLE patients, which suggest that oxidative modification act as biomarkers. Increased MDA (malondialdehyde)-modified proteins, anti-SOD and anti-catalase antibodies, albumin modification by HNE (4-hydroxy 2-nonenal) in the sera of SLE patients are associated with disease activity in SLE patients [11,12]. Levels of F2 isoprostane (8-iso-PGF2), a derivative of lipid peroxidation, increased in urine from SLE patients and are associated with disease activity [13]. Many independent studies show the elevated levels of MDA, F2-Isoprostane, nitric oxide and diminished levels of reduced glutathione in patients with lupus nephritis [14-17]. The elegant research from Frosegard group has shown the elevated levels of oxidized low-density lipoprotein (OxLDL) together with elevated levels of autoantibodies as risk factors for cardiovascular disease in SLE patients [18]. Table 1 summarizes various studies of oxidative stress biomarkers in SLE. These biomarkers are important for predicting the consequences of oxidation and providing a basis for designing appropriate interventions to prevent or alleviate an injury. Recently, there has been a great improvement in assay methods and measurement accuracy for biomarkers of oxidative stress, which have been correlated with disease activity and progression of disease, however this has not been validated in the clinic.

**Table 1:** A summary of reported oxidant and antioxidant biomarkers in SLE

**Study**	**ROS**	**Lipid peroxidation**	**SOD**	**Catalase**	**Glutathione peroxidase**	**GSH**	**Nitric oxide**	**Protein oxidation**	**DNA oxidation**	**Tissue/Cell studied**
Shah et al. [14,19-22]	↑	↑	↓	↓	↓	↓				RBC, Serum, Lymphocyte
Perl et al. [23,24]	↑	↑				↓				Lymhocyte
Turi et al. [25]		↑	↓	↓	↓	↓				RBC
Hassan et al. [26]		↑			↓	↓				Serum
Kurient & Scofied [4,27]		↑	↓							Serum
Taysi et al. [28]		↑		↓	↓					Serum
Serban et al. [29]		↑			↓	↓				RBC, Plasma
Turgay et al. [30]		↑	↓	↑	↓					Plasma
Segal et. al. [31]		↑								Plasma
Bae et al. [32]		↑	↓		↓					Plasma
Jovanovic et al. [13]		↑								Plasma
Abou-raya et al. [33]		↑								Serum
Vipartene et al. [28]		↑	↓		↓					RBC
Mohan & Das [34]		↑	↓		↓					Plasma
Tewthanan et. al. [16,35]		↑				↓				Plasma
Morgan et. al. [36,37]			↓	↓	↓	↓		↑		Serum
Zhang et al. [38,39]		↑						↑		Serum, Blood
Ahsan et al. [40]								↑		Serum
Lunec et al. [41]									↑	Urine
Evan et al. [42]									↑	Serum
Maeshima et al. [43]									↑	Urine
Ho et al. [44]							↑			Plasma
Gilkeson et al. [17]							↑			Serum
Wanchu et al. [45]							↑			Serum

↑, significantly elevated levels; ↓, significantly diminished levels.

This review examines the available evidence for the involvement of cellular oxidants in the pathogenesis of SLE and the current biomarkers of oxidative stress focusing on their association with disease complication, which may be useful for developing ideal biomarkers in disease.

## Review

### Reactive oxygen and nitrogen species

ROS is a collective term for the chemical species that are formed as a result of incomplete reduction of oxygen. It includes superoxide anion radical (O_2_^**–.**^), peroxy radical (ROO^**−**^), hydrogen peroxide (H_2_O_2_), singlet oxygen (^1^O_2_), perhydroxyl radical (HO_2_^**.**^) and extremely reactive hydroxyl radical (^**.**^OH). ROS are short-lived molecules produced by normal cellular metabolism that are well recognized for playing a dual role; they are both deleterious and beneficial species. At low or moderate concentrations, ROS is required for the regulation of many cellular processes, including cell signaling, differentiation, proliferation, growth, apoptosis, and cytoskeletal regulation, and can act as lethal weapons for the host defense system. The harmful effect of free radicals occurs when there is an overproduction of ROS/RNS or a deficiency of enzymatic and non-enzymatic antioxidants. Reactive intermediates are either produced by reactions involving enzymes such as nicotinamide adenine dinucleotide phosphate (NADP)H [6], nitric oxide synthase, or by nonenzymatic reactions through mitochondrial electron transport chain [46], and reduced transition metals [47]. ROS can also interact with nitric oxide (NO), the product of NO synthases, whose expression is usually accompanied by inflammatory lesions, resulting in the conversion of NO to various reactive nitrogen species (RNS^**.**^), which include nitrosonium cation (NO^+^), nitroxyl anion (NO^−^) and peroxynitrite (ONOO^**.**^). Many independent studies show a significant correlation between global lupus disease activity and markers of systemic NO production [17].

### Sources of reactive oxygen species and their scavengers

Reactive oxygen species mostly originate from mitochondria, blood cells (lymphocytes, RBC) and vascular endothelial cells in patients with SLE and these ROS cause hyperpolarization of mitochondria, activated T lymphocytes, apoptosis and endothelial activation [23,48]. Several studies have shown increased production of ROS or diminished levels of intracellular reduced glutathione in various blood components in SLE patients [19,24]. In addition, ROS can be produced by other sources like NADPH oxidase (NOX enzyme) in activated phagocytes [49] and to a lesser extent in macrophage and polymononuclear cells [50,51], lysosome (myeloperoxidase undergoes a complex array of redox transformations and produces HOCl) and microsomes [52,53]. Hydroxyl radicals are generated from peroxynitrite, which is in turn rapidly formed through the reaction between NO^•^ and O_2_^–•^ under appropriate stoichiometric conditions. Hydrogen peroxide is formed through the dismutation of O_2_^–•^ catalyzed by the enzyme superoxide dismutase, and is also produced via. action of several other oxidase enzymes (e.g. aminoacid oxidases). Tissue inflammation and chronic infection lead to the overproduction of ^**·**^NO and O_2_^–•^, which rapidly combine to yield peroxynitrite: O_2_^•–^ + ^•^NO → ONO_2_^•−^. In addition, ROS may amplify the inflammation process of gene expression involved in the inflammatory response, particularly via. activation of the nuclear transcription factor NF-kβ, which in turn upregulates pro-inflammatory cytokines and leukocyte adhesion molecules. Exogenous sources of ROS include; radiation, UV light, x-rays, gamma rays, chemicals that react to form peroxides, ozone and singlet oxygen, chemicals that promote superoxide formation, quinones, nitroaromatics, bipyrimidiulium herbicides, chemicals that are metabolized to radicals e.g., polyhalogenated alkanes, phenols, aminophenols etc. [54,55].

**Figure 1:**
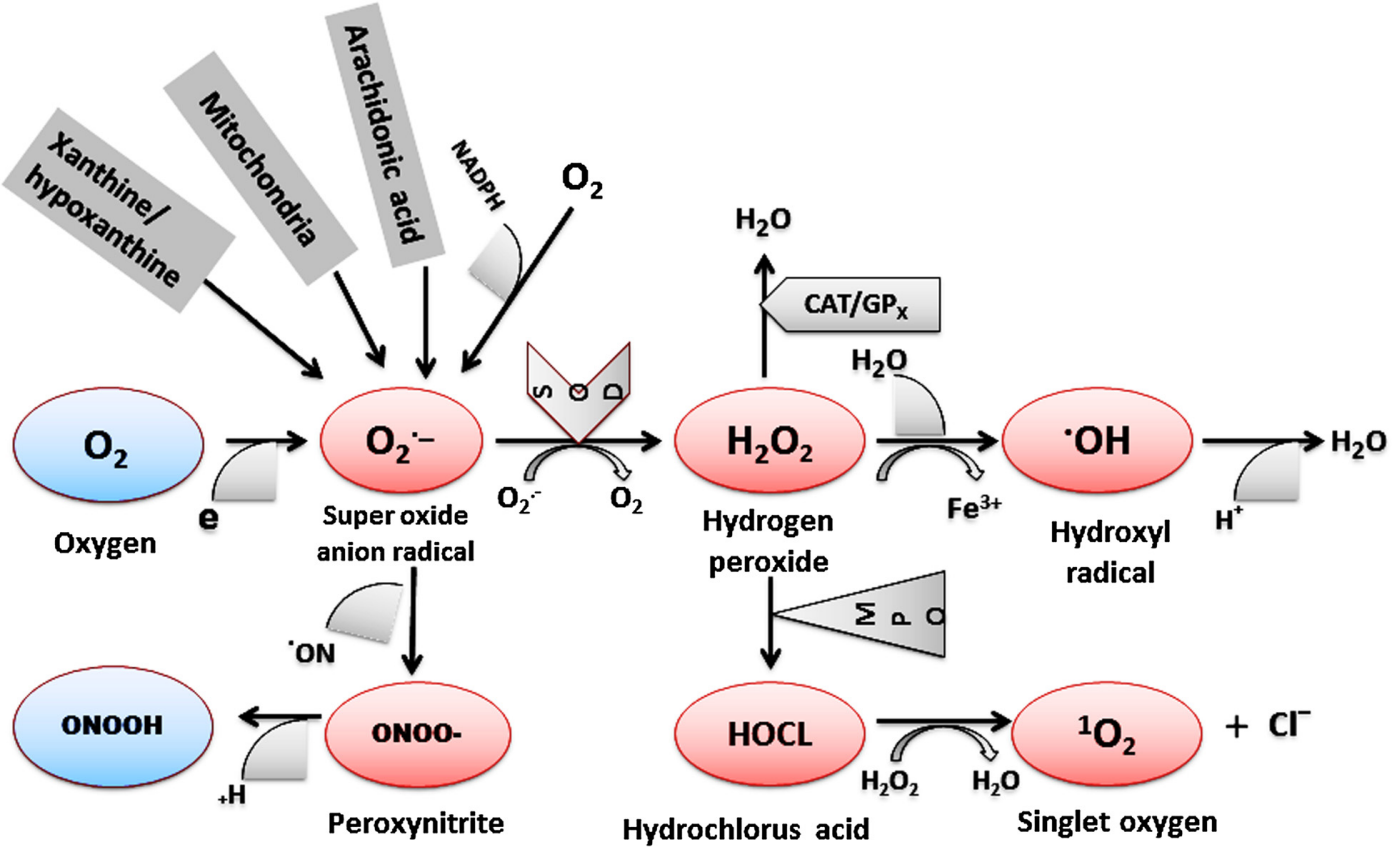
**Main sources of free radical generation and their catabolism.** Highly reactive superoxide anions are mainly produced in mitochondrial, xanthine oxidase and NADPH-oxidase. Superoxide anions can either react with nitric oxide to generate the strong oxidant peroxynitrite, or be degraded by superoxide dismutase into the less reactive species hydrogen peroxide. Hydrogen peroxide can then be catabolized by glutathione peroxidase or catalase reaction, react with Fe^2+^ to form hydroxyl radicals via the Fenton reaction, or be degraded by the myeloperoxydase, another source of hydroxyl radicals.

Most damaging ROS are the hydroxyl radical, OH^–•^ and O_2_^–•^; the latter can be converted into relatively stable, nonradical hydrogen peroxide by superoxide dismutase enzyme and then hydrogen peroxide is reduced by three general mechanisms (Figure 1). First, it is the substrate for two enzymes, catalase and glutathione peroxidase, which catalyze the conversion of H_2_O_2_ to H_2_O + O_2_; this presumably is a detoxification mechanism. Secondly, H_2_O_2_ is converted by myeloperoxidase (MPO) in neutrophils to hypochlorous acid (HOCl). This appears to be a mechanism for a physiological toxic agent, since HOCl is a strong oxidant that acts as a bactericidal agent in phagocytic cells. Reaction of HOCl with H_2_O_2_ yields singlet oxygen (^1^O_2_) and water. The biological significance of singlet oxygen is unclear. Thirdly, H_2_O_2_ is converted in a spontaneous reaction catalyzed by Fe^2+^ (Fenton reaction) to highly reactive hydroxyl radical (^•^OH). As a hydroxyl radical cannot be eliminated without causing oxidative damage, it reacts promptly with any biological molecule like lipid, protein and DNA, which causes severe consequence in the pathogenesis of SLE [27,36,56,57].

### Interaction of reactive oxygen species with lipids, proteins and DNA

**Figure 2:**
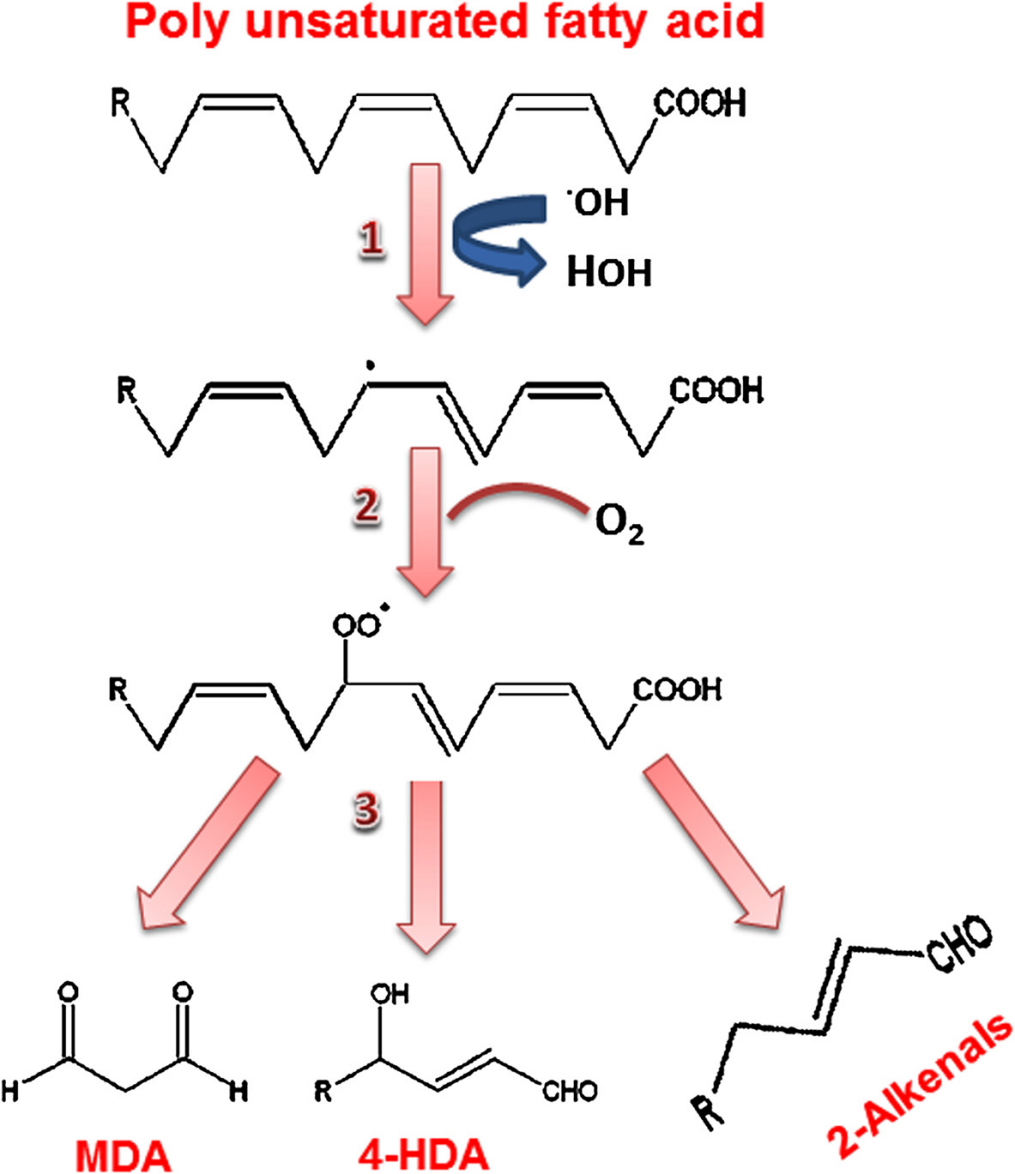
**Major aldehydic products of lipid peroxidation.** ROS, in particular hydroxyl radical, react with lipid membranes and generates reactive aldehydes including malondialdehyde (MDA) and 4-hydroxy-2-nonenal (HNE), in three phage reactions.

ROS, in particular the hydroxyl radical, react with lipid membrances and generate reactive aldehydes including MDA and HNE, in three phase reactions (Figure 2), which can ‘spread’ oxidative damage through the circulation in SLE [58]. In the initiation phase, a primary reactive radical, abstracts a hydrogen atom from a methylene group to start peroxidation. This results in the formation of a conjugated diene, leaving an unpaired electron on the carbon. The carbon-centered fatty acid radicals combine with molecular oxygen, in the propagation phase, yielding highly reactive peroxyl radicals that react with another lipid molecule to form hydroperoxides. Peroxyl radicals are capable of producing new fatty acid radicals, resulting in a radical chain reaction. The cascades of lipid peroxidation result in a variety of harmful end products include conjugated dienes, isoprostanes, HNE, HNE-modified proteins, MDA, MDA-modified proteins, protein-bound acrolein and oxHDL which are associated with disease activity in SLE [59-61]. In addition to the involvement of ROS in lipid peroxidation, ROS can modify both the structure and function of proteins [36,62]. Metal-catalyzed protein oxidation results in the addition of carbonyl groups or cross-linking or fragmentation of proteins. Lipid (peroxidation) aldehydes can react with sulfhydryl (cysteine) or basic amino acids (histidine, lysine). Similarly, modification of individual nucleotide bases, single-strand breaks and cross-linking are the typical effects of ROS on nucleic acids. All these interactions of ROS with protein, lipid and nucleic acid are involved in auto-antibody formation in SLE patients [13,63]. Table 2 summarizes the important studies on antibodies formation against modified lipid, protein and DNA in SLE patients.

**Table 2:** A summary of reported antibodies against antioxidant enzymes and oxidatively modified proteins in SLE

**Study**	**Anti-SOD antibody**	**Anti-catalse antibody**	**Anti-oxidised antibody**	**Anti-MDA or HNE adduct**	**MDA/HNE modified protein**	**Anti-lipid antibody**	**Tissue/Cell studied**
Mansour et al. [11,12]	↑	↑			↑		Serum
Kurien & Scofield [4]	↑						Serum
Wang et al. [64]				↑	↑		Serum
Jovanovic et al. [13]						↑	Plasma
Toyoda et al. [65]			↑				Serum
Lopez et al. [18]						↑	Serum

↑, significantly elevated levels; ↓, significantly diminished levels.

### Antioxidant defense system

The damaging effect of ROS is limited by the numerous cellular antioxidant defense mechanisms in the body. Common antioxidants include: enzymes such as SOD, CAT and glutathione related enzymes (GPx, GR, GST and thioredoxin reductase) and heme oxygenase [66], non-enzymes such as vitamins (A, C, E) and carotenoids, flavonoids, glutathione and other antioxidants minerals (copper, ferritin, zinc, manganese, selenium etc.) [67,68]. They work in synergy with each other against different types of free radicals. Vitamin E suppresses the propagation of lipid peroxidation, while vitamin C with E impedes hydroperoxidase formation [29]. Antioxidant defences are mainly dependent upon *de novo* synthesis of thiol proteins (glutathione and thioredoxin) and diminished levels of the antioxidant molecule glutathione have been associated with various clinical complications in SLE.

### Glutathione

Glutathione (L-γ-glutamyl-L-cysteinylglycine) is required for many critical cellular processes and plays a particularly important role in the maintenance and regulation of the thiol-redox status of the cell. In healthy cells and tissues, more than 90% of the total glutathione pool is in the reduced form (GSH) and less than 10% exists in the oxidized form (GSSG) [69]. The GSH/GSSG ratio is a valuable tool for defining oxidative stress and changes in this ratio appear to correlate with disease activity in SLE patients [19,62]. Diminished level of intracellular glutathione has been associated with immune dysfunction (T cell activation, imbalance Th1/Th2 cytokines and deregulation of apoptosis) and organ damage (nephritis, CNS) in SLE [20,70,71]. Furthermore, replenishment of the intracellular glutathione has been associated with diminution of autoantibody levels and the development of nephritis and prolong mice survival [72] while in SLE patients, it has been associated with improved disease activity and fatigue [24,35]. These studies conclude that depletion of intracellular glutathione is an indicator of oxidative stress in SLE and replenishment of intracellular glutathione may attenuate disease complications.

### Superoxide dismutase

Superoxide dismutase is a metalloprotein, considered to be the first line of defence against free radical formation. It catalyzes dismutation of superoxide radical into oxygen and hydrogen peroxide. Superoxide radical, if not scavenged effectively, may directly inactivate several enzymes like CAT and GPx which are needed to eliminate hydrogen peroxide from intracellular medium. Three forms of this enzyme found in human are: SOD1 located in cytoplasm, SOD2 in mitochondria and SOD3 in extracellular [73]. SOD1 is a dimeric, while SOD2 and SOD3 are tetrameric. SOD1 and SOD3 contain copper and zinc, while SOD2 has manganese in its reactive center. Several groups have reported a decreased activity of SOD and formation of auto-antibody against SOD enzyme in SLE patients [14,21,30]. It is speculated that the antibody to SOD is responsible for inactivation of enzyme and exaggerated oxidative damage in SLE.

### Catalase

Catalase, located in peroxisomes (80%) and cytosol (20%), decomposes hydrogen peroxide to water and oxygen without the production of free radicals [74]. Concentration of CAT is highest in liver, kidney and erythrocyte and low in connective tissues [75]. In tissues, it exists mainly as particle bound (mitochondria and peroxisomes), while in erythrocytes it exists in soluble state. Catalase does not show significant activity under physiological conditions due to its lower affinity than glutathione peroxidase for hydrogen peroxide, but becomes an important enzyme at disease state where concentration of H_2_O_2_ is elevated [47]. CAT polymorphism (−330CC genotype) showed a significant association with thrombocytopenia, renal manifestations, as well as production of anti-snRNP and anti-Scl-70 antibodies in SLE patients [8]. An elegant study from Mansour group showed elevated levels of auto-antibodies against catalase in SLE patients. In two different clinical studies, they have demonstrated that SLE patients have increased levels of IgG antibodies against catalase which are associated with disease complication [11,12].

### Glutathione peroxidase and glutathione reductase

Glutatione peroxidase and reductase are glutathione dependent enzymes located in the cytoplasm, mitochondria and nucleus [76]. Glutatione peroxidase metabolizes hydrogen peroxide to water using reduced glutathione as a hydrogen donor and is recycled back to glutathione reductase by cofactor NADPH. It plays an important role in the defense mechanism in the erythrocytes against lipid peroxidation damage [77]. In SLE patients, decreased activity of GPx and GR may lead to altered redox state [30].

### Biomarkers of oxidative stress

The production of free radicals is an integral part of metabolism, which if unchecked, cause oxidative stress. Oxidative stress damage to lipid, protein, nucleic acids and carbohydrates are deleterious and concomitant. There are substantial accumulation of data suggest that oxidative stress biomarkers can not only determine the extent of oxidative injury, but also indicate the source of oxidant [5]. Biomarkers of oxidative stress are also important for predicating the consequences of oxidation and for providing a basis for designing appropriate intervention to prevent or alleviate injury. The most intuitive goals for a biomarker are to help the diagnose symptomatic and pre-symptomatic disease and to provide surrogate endpoints to demonstrate clinical efficacy of new treatments. In the diverse nature of disease like SLE, single biomarker analysis can not reflect the whole body oxidative damage and a series of biomarkers may be required and each needs to validate in prospective clinical studies. A valid biomarker should be [78,79]:

1. A stable product of oxidative stress, not susceptible to artificial induction or loss during storage.

2. Detectable in the target tissue or a valid surrogate tissue where it causes oxidative modification and damage.

3. Present in sufficient and measurable concentrations.

4. Determined by an assay that is specific, sensitive, reproducible, and robust.

5. Free of confounding factors from dietary intake.

6. Measurable within a detection limit of a reliable analytical procedure.

**Figure 3:**
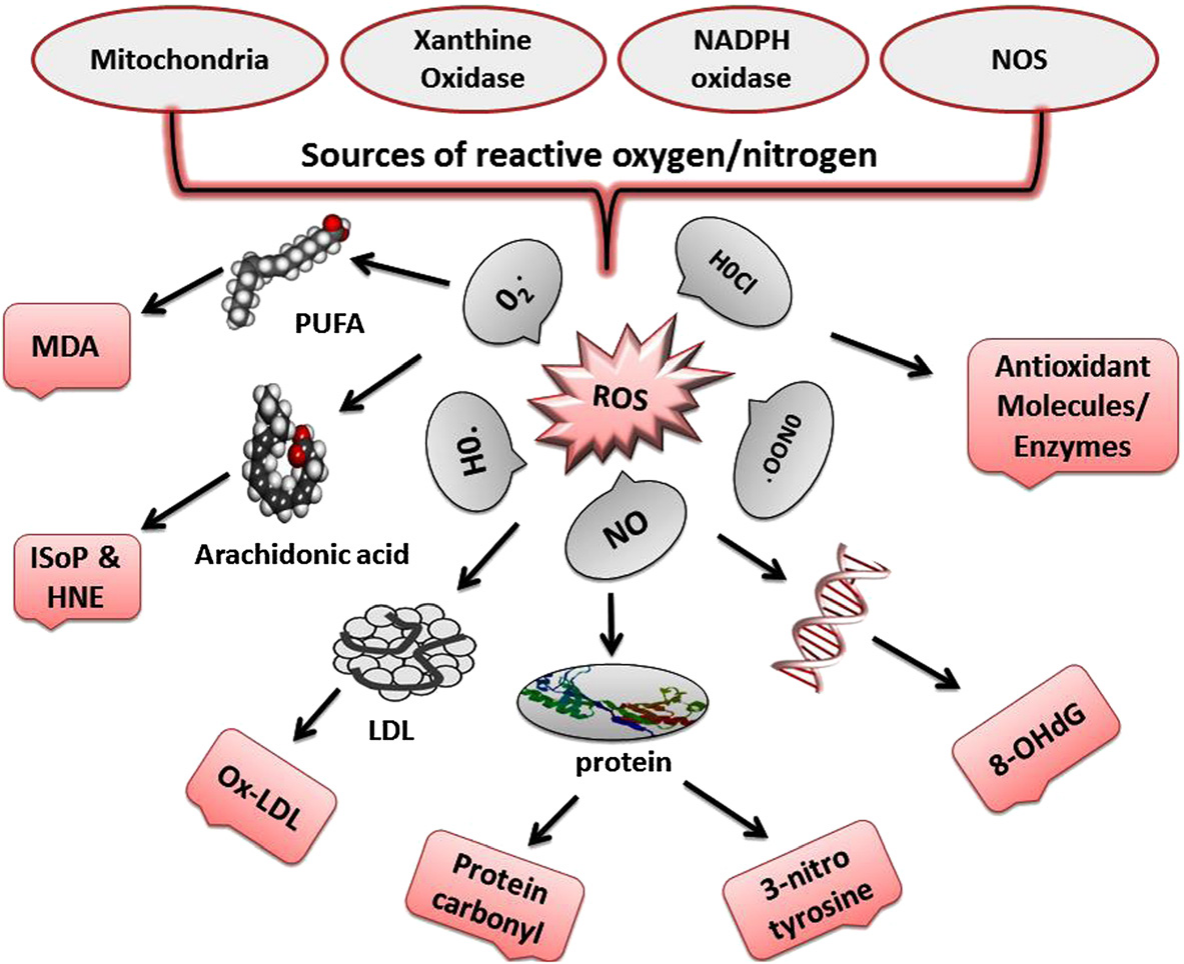
**Formation of oxidative modified biomarkers by reactive oxygen species.** Lipid peroxidation biomarkers: malondialdehyde, F2-isoprostane, acrolein and Ox-LDL. Protein oxidation markers: protein carbonyl and protein nitration. Oxidative DNA damage biomarkers: 8-hydroxy-2′-deoxyguanosine (8-OHdG). Antioxidant enzymes and molecules: superoxide dismutase, catalase, glutathione peroxidase, oxidized glutathione, total antioxidant capacity.

Availability of biomarkers that provide an accurate assessment of the degree of oxidative stress are important in clinical trials aimed at investigation of the effectiveness of antioxidant therapy for preventing or alleviating complications. Recently, there has been a great improvement in assay methods and measurement accuracy for biomarkers of oxidative stress in SLE patients and animal models. Table 3 summarizes the selected oxidative stress biomarkers and their methods of detection. ROS/RNS has been directly detected *in vitro* by electron spin resonance with or without spin-trapping reagents or by chemiluminescence. These direct detection methods have not yet applied for clinical examination because of the instability of many reactive species (half-life even much shorter than second) and the requirement of sophisticated techniques. However, recently 2′,7′-dichlorofluorescein (H2DCF) and Dihydroethidium (DHE), have been used extensively to evaluate ROS production in various samples in SLE patients by flow cytometer [19,80]. The end products/metabolites of ROS/RNS are stable, can accumulate to detectable concentrations, reflect specific oxidation pathways, and correlate with disease severity. These are shown schematically in Figure 3. Of these modifications, some are known to have direct effects on function of molecule (e.g. inhibit antioxidant enzyme function), but others merely reflect the degree of oxidative stress in the local environment. These metabolites include lipid peroxidation end products (malondialdehyde, F2-isoprostane, HNE, acrolein and Ox-LDL), oxidized proteins (protein carbonyl and protein nitrotyrosine), DNA oxidation (8-OHdG), nitric oxide and antioxidant enzymes (SOD, CAT, GPx, GR and total antioxidant capacity [17,44,45].

**Table 3:** List of oxidative stress biomarkers and their methods of detection

**Free radicals and reactive species**			**Markers/probes and detection methods**
**ROS/RNS**	**Direct measurement**	ROS	1. Electron spin resonance (ERS) – Spectroscopy method
			2. Fluorescence (2′,7′ dichlorofluorescein) - Flow cytometry/spectroflurometry
		RNS	1. Ion electrode method
	**Indirect measurement**	Lipid	1. Malondialdehyde (MDA) – colorometric, flurometric, ELISA, HPLC
			2. Thiobarbituric acid reactive substances (TBARS) - colorimetric, flurometric
			3. 4-Hydroxynonenal (4-HNE) – GC- MS, HPLC
			4. F2 isoprostanes (8-iso-PGF2) – colorimetric, flurometric, ELISA
			5. Lipid hydroperoxidation - Hexanoyl - Lys adduct (HEL) - ELISA, HPLC
			6. Oxidized low density Lipoprotein – HPLC, ELISA
		Protein	1. Carbonyl – colorometric, ELISA
			2. 3-nitrotyrosine - GC-MS, HPLC, ELISA
			3. Protein Thiol - colorimetric, ELISA
		DNA	1. 8-Hydroxy-2′deoxyguanosine (8-OHdG) - HPLC, LC, MC, ELISA
			2. DNA Break - comet assay, flow cytometry
**Antioxidant**	Antioxidant enzyme activity	Super oxide dismutase, Glutathione Peroxidase, Catalase, Glutathione reductase, Xanthine oxidase- ELISA, colorimetric method	
		Glutathione - HPLC, colorimetric/flurometric method	
	Antioxidant non-enzymes	Ascorbic acid, α tocopherol, β carotene, Lycopene – colorimetric Zinc, Selenium, Mn, Cu, Fe – Flame Photometrry	
**Antioxidant capacity**	1. Total Antioxidant Status (TAS) – colorimetric method		
	2. Triolox Equivalent antioxidant Capacity (TEAC) – colorimetric method		
	3. Ferric Reducing Antioxidant Power (FRAP) – colorimetric method		
	4. Asymmetric Dimethylarginie (ADMA) – colorimetric method		

### Biomarkers of lipid damage

Lipids are susceptible targets of oxidation, and lipid peroxidation products are potential biomarkers for oxidative stress status in SLE [34,81]. Lipid peroxidation generates a variety of relatively stable decomposition end products, mainly unsaturated reactive aldehydes, such as MDA, Hexanoyl-Lys adduct (HEL), HNE and 2-propenal (acrolein) [82], and isoprostanes [83], which can be measured in various biological samples (serum/plasma and urine) as an indirect index of oxidative stress. Three of the most well studied markers of lipid peroxidation are MDA, HNE and 8-isoPGF_2_ in SLE patients and animal models, though some others lipid peroxidation markers (acrolein, OxLDL, oxidized phospholipid/apolipoprotein-B) have been reported in few studies [15,18].

### Malonaldehyde

MDA is generated *in vivo* by peroxidation of polyunsatuated fatty acids and represent a stable end product of lipid peroxidation. It is an extensively studied biomarker of lipid peroxidation in SLE patients and animal models due to the simple method of detection. MDA is typically quantified from various blood compartments (plasma, serum, lymphocytes) and tissue using a colorimetric assay based on the reaction between MDA and thiobarbituric acid (TBA). This is simple and is the most frequently used method in lipid peroxidation research, but other (aldehydes) compounds also react with TBA to form color that can interfere with this assay. Other more sensitive methods like HPLC, LC-MS and MS-MS can separate MDA from other aldehydes and this is suggested as a sensitive technique for measuring levels of MDA in various biological fluids in SLE patients, though some scientists question its clinical utility. Recently, a high sensitive ELISA method has been developed for measuring MDA levels in serum/plasma or other biological fluids. This antibody based method is typically validated against the measurement of MDA by HPLC and it demonstrates better performance with improved specificity [84]. Increased levels of MDA have been associated with many clinical features like lupus nephritis and tissue damage in SLE [14,25]. Several groups have shown an increased level of MDA and its association with nephritis and CVD in SLE patients [14,25,26]. The clinical trials attempting to replenish intracellular glutathione using N-acetyl cysteine (NAC) (24 SLE patients, Perl group and 40 SLE patients, Tewthanom group) have shown to reduce MDA levels and lupus nephritis [24,35]. These studies suggest a potential role of lipid oxidation in prediction the progression of nephritis and response to therapy. However, the mechanism of the action of NAC is currently under investigation and further studies are required to determine the outcome of NAC treatment with SLE patients.

### 4-hydroxy-2-nonenal

HNE is a major and toxic aldehyde generated by free radical attack on polyunsaturated fatty acids (arachidonic, linoleic, and linolenic acids) and is considered a second toxic messenger of oxygen free radicals [85]. It is enduringly formed at basal concentrations under physiologic conditions, but its production is greatly enhanced in lipid peroxidation condition. Level of HNE can be measured by methods like HPLC, GM-CS, however, ELISA is the most used method in SLE patients due to the simplicity of the assay. Increased levels of HNE have been demonstrated in the MRL/lpr as well as in SLE and associated with increased onset of disease [86,87]. Increased HNE has been shown to exhibit facile reactivity with various biomolecules, including proteins and DNA, and is regarded as a sensitive marker for evaluating oxidative stress in disease state [65,87]. However, a longitudinal study may be helpful to understand its association with clinical feature in SLE patients.

### F2-Isoprostanes

F2-Isoprostanes are a group of bioactive prostaglandin-like compounds generated by free radical mediated peroxidation of arachidonic acid *in vivo*[88]. Sources of free radicals for isoprostanes formation include: i) mitochondrial electron transport chain (O_2_^**.**−^ and ^**.**^OH), ii) P450 enzymes (O_2_^**.**−^ and ^**.**^OH), iii) lipoxygenase (hydroperoxyl radical (HO_2_^**.**^) and iv) transition-metal catalysed formation of free radicals [64,89]. F2-Isoprostanes especially 8-iso-PGF2-alpha has been proposed as specific, reliable, and non-invasive markers of lipid peroxidation *in vivo* in various biological fluids among which plasma and urine are the most commonly used samples [33]. Unlike MDA, levels of F2-Isoprostanes are not influenced by lipid content in diet. Available data indicate that quantification of F2-Isoprostanes in either plasma or urine gives a highly precise and accurate index of oxidative mediated vascular involvement in SLE patients [33]. Measurement of F2-Isoprostanes may be useful to understand the causative role of oxidative damage in vascular disease in SLE. Mass chromatography technique (GC-MS) and ELISA can accurately and sensitively measure F2-Isoprostanes in biological samples. ELISA is a frequently used technique to quantify F2-Isoprostanes because of its low cost and ease of use [90]. Levels of F2-Isoprostanes in plasma and urine samples have been shown to correlate with *in vivo* oxidative stress in a number of studies in SLE patients. Studies from Avalos (95 SLE patients), Segal group (71 SLE patients) and Abou-Raya (60 SLE patients) have shown that F2 isoprostane levels are associated with increased disease activity, fatigue and lower quality of life [31,33,91].

Among all the available markers of lipid peroxidation, F2-Isoprostanes is well suited as a biomarker for the following reasons: (i) *In vivo* formation of isoprostane increases as a function of lipid peroxidation. (ii) It is stable and can be easily measured with high accuracy and is not influenced by lipid content in the diet. (iii) It is present in detectable amounts in all healthy tissues and biological fluids, thus allowing definition of a reference interval [79]. Besides these biomarkers, some other biomarkers of lipid peroxidation like, OxLDL, oxidized phospholipid/apolipoprotein-B have been reported to be associated with arterial disease and renal manifestations in SLE patients. Also, NAC treatment has shown to improve cardiovascular and renal outcomes in these patients [15,18,24].

### Biomarkers of protein damage

Oxidative modification of proteins is known to affect protein function. Protein carbonyls and protein nitrotyrosine are widely used and chemically stable biomarkers of protein oxidation in SLE [92].

### Protein carbonyls

The protein carbonyls group are formed by either direct oxidation of certain amino acid residues, particularly lysine, arginine, threonine, proline and histidine or secondarly reaction with product of lipid peroxidation (e.g., HNE) or glycoxidation reaction with lysine group [79]. Protein carbonyls are better studied than protein nitration in SLE patients. They circulate for longer periods in blood as compared to other oxidized product, can be stored for a long time, which make them suitable markers for protein oxidation. Several studies have shown an elevated level of total protein carbonyls in SLE patients and exhibited varying correlations with disease activity [36-38]. It can be measured by spectrophotometer, HPLC, Western blot and ELISA [93]. However, these methods cannot identify which amino acid residues are oxidatively attacked and which protein has been modified. Furthermore, the specific association of protein oxidation and organ damage in SLE patients needs further longitudinal study to establish a relationship to use as a promising biomarker.

### Protein nitrotyrosine

Protein nitrotyrosine is generated by RNS species like peroxynitrite (ONOO^−^) and nitrogen dioxide (NO_2_) on tyrosine residues of protein. Many independent studies show increased levels of 3-nitrotyrosine and which is associated with arthritis, cardiac and renal involvement in SLE patients [39,40]. The 3-nitrotyrosine can be measured by antibody based ELISA method as well as by GC-MS and LC-MS with more precision. However, more efforts are needed to improve the methodology for the measurement of 3-nitrotyrosine in particular when concentration is very low and further studies are required to appraise the association of circulating nitrated proteins and disease complication like cardiac and renal in SLE patients.

### Biomarkers of DNA damage

Free radicals may also bring about the oxidative damages of DNA that are manifested by the development of various complications in SLE patients [43,93]. The most commonly used marker of oxidatively modified DNA is 8-hydroxy-2′-deoxyguanosine (8-OHdG), a product of oxidatively modified DNA base guanine [42,93,94]. Biological materials most often used to measure levels of 8-OHdG include, serum, plasma, urine and tissues. Levels of 8-OHdG in various biological samples (specially urine) has been correlated with disease activity in SLE patients [41], thus 8-OHdG is a useful marker for study of DNA damage caused by free radicals. Many methods such as HPLC, GC-MS, LC-MS and ELISA have been available to measure levels of 8-OHdG in the biological samples and are reviewed in detail in several articles [95-97]. HPLC is a frequently used method with high accuracy and sensitivity, but the procedure is complex and time-consuming making it less promising compared to ELISA in clinical use [97,98]. Measurement of urinary 8-OHdG has been found to reflect the whole body oxidative damage [78,98] and was independent of dietary influence in human [99].

Besides above oxidative stress markers of biomolecules (lipid, protein and DNA), antioxidant enzymes/molecules have been regularly used to evaluate the antioxidant defense system in human body [100]. Among these antioxidant enzymes (SOD, CAT, GPx, XO)/molecules (ascorbic acid, β-carotene, Zn, Selenium, Cu, Fe), glutathione redox (GSH/GSSG) has been shown to be a reliable marker for whole body antioxidant index in disease [28,32,101,102]. It has been well established that a decrease in GSH concentration may be associated with the pathogenesis of SLE [19,22,62]. Several methods have been optimized to measure glutathione forms in human samples, including colorimetric, HPLC and GC-MS. Even after 3 decades of incredible research on oxidative stress in SLE, no biomarker of oxidative stress is currently accessible for clinical use, however there are some markers, glutathione, HNE, F2 isoprostane, 8-OHdG are emerging as reliable markers for measuring oxidative stress and reflecting disease activity in SLE patients.

### Antioxidant therapy for SLE

As described in the preceding sections, imbalance between oxidant and antioxidant enzymes in favor of the former and contributes to the pathogenesis of SLE. Graceful research from various investigators suggest that restoration of the redox balance using antioxidant agents (NAC) or diminishing effect of oxidative stress by intake of antioxidant nutrients, vitamins A, C and E, carotene, lycopene etc., may attenuate various oxidative stress induced complication in SLE [24,34]. Among all the antioxidant treatment to restore the redox balance, NAC has shown to be a promising alternate therapy in both SLE patients and animal models. Murine models of lupus showed that NAC treatment suppressed autoantibody formation, reduced nephritis and prolonged survival [72]. Several groups have shown that the decrease in intracellular glutathione has been associated with many clinical features like, nephritis [14], CNS [103], endothelial activation in cardiovascular and cerebrovascular involvement [104] in SLE patients. Administration of NAC has shown beneficial effect in mild SLE patients in terms of decreasing lipid peroxidation, improve CNS complication [103], endothelial function [104,105] in patients with cerebrovascular involvement. Moreover, it has been shown that NAC treatment control T cell function by regulating rapamycin (mTOR) mechanism [106]. Other antioxidant like cystamine (CYST) has shown to be beneficial in the treatment of (NZW × NZW)F1 lupus-prone mice, however CYST in the treatment of SLE patients are lacking [107]. Collectively, therapeutic interventions that replenish the redox balance or decrease exposure to ROS and/or augment antioxidant defenses might be beneficial adjunctive therapy in the treatment of oxidative damage in SLE.

## Conclusions

Oxidative stress biomarkers may have a role in the future to assist clinical decisions regarding the use of antioxidant therapies and their efficacy. There has been great progress in the development of oxidative stress biomarkers, but due to the complex nature of disease, there is an extremely low possibility that a single biomarker can reflect the whole body of oxidative damage and its role in the pathophysiology of disease. Instead a series of biomarkers may be required and each needs to be validated in prospective clinical studies. It might also be essential to determine which particular marker, alone or in combination with others, can serve as true indicator of the contribution of oxidative stress to a disease. Methodologies need further improvement, so that they can be validated among patients and apposite for clinical use. Pilot clinical study on SLE patients showed the replenishment of intracellular glutathione using NAC reduce oxidation of lipid, protein and DNA and improved organ damage in SLE patients. However, future controlled clinical studies are required to test the broad spectrum of the NAC effect and its mechanism of action in combination with conventional therapy in SLE patients. Moreover, the measurement of biomarkers with the intention of accurate determination and their association with the improvement of disease during antioxidant therapy may open the new door for the management of disease.

## Competing interests

The authors declare that they have no competing interests.

## Authors’ contributions

DS and SS have prepared manuscript. DS, NM, BP and SS collected information and prepared tables and figures. DS and NSK have done overall supervision. All authors read and approved the final manuscript.
